# Effect of Laser Speed on Cutting Characteristics of Cement-Based Materials

**DOI:** 10.3390/ma11071055

**Published:** 2018-06-21

**Authors:** Dongkyoung Lee, Youngjin Seo, Sukhoon Pyo

**Affiliations:** 1 Department of Mechanical and Automotive Engineering, Kongju National University, Cheonan 31080, Korea; ldkkinka@kongju.ac.kr (D.L.); syjvlfry1004@gmail.com (Y.S.); 2 Korea Railroad Research Institute, Uiwang-si 16105, Korea

**Keywords:** cement-based materials, laser cutting, chemical analysis, multi-mode fiber laser, high-temperature properties

## Abstract

The results of an experimental investigation on the physical and chemical characteristics of cement-based materials under laser interactions are presented. The laser cutting tests were conducted using a multi-mode continuous fiber laser with a laser power of 1 kW. The experimental variables were laser speed, water to cement ratio, and material compositions including cement paste, cement mortar, and ultra high-performance concrete (UHPC). In order to evaluate the mass removal mechanisms of cement-based materials under laser interactions, the effect of laser cutting was evaluated in terms of kerf width, penetration depth, and chemical composition changes before and after the interaction with laser using EDX analysis. The test results reveal that adding silica sand in cement-based materials leads to decreasing penetration depth and increasing kerf width. Unlike the cement paste and cement mortar series, UHPC specimens showed no discernible crack observed by the naked eye after laser interaction due to its high strength. Furthermore, the chemical analysis indicates that chemical composition changes were caused by various mechanisms including dehydration of calcium hydroxide and thermal decomposition of calcium carbonate.

## 1. Introduction

Conventional cutting of cement-based materials has disadvantages such as degradation of accuracy, fallout, a need for close operator proximity, and the creation of considerable amounts of effluent [1]. Laser-aided manufacturing (LAM) has been used in many applications [2,3,4,5,6,7,8,9,10,11,12,13,14,15,16,17,18], since it has many advantages (e.g., a low heat affected zone (HAZ), higher precision levels, faster speed, and a non-contact method). The advantage of the non-contact method is that it provides almost no tool wear, thus there is no need for tool replacement, so LAM can be used almost permanently. Moreover, LAM can provide high-energy density and high-processing speed. In addition, from a practical point of view, a laser providing high-energy density can also be applied to remove a large volume of cement-based construction materials, as high-power laser beams are commercially available, and the laser beam can be focused on very small areas by manipulating optics.

Due to these advantages, LAM has been used to treat or the concrete surfaces. Savina et al. [19] used a pulsed Nd:YAG laser with fiber optic beam delivery to ablate surface and near-surface regions of concrete. They used the 1064 nm fundamental of an Electrox 1.6 kW pulsed Nd:YAG laser with a fiber optic cable and focused to a 0.55 mm spot via a 120 mm lens. The sample stage was moved at a rate of 45 cm/s while the laser was fixed. They found that cement matrices melts, dehydrates, and vaporizes, while silica-rich aggregate tends to fracture and dislodge without melting with laser interaction. Lawrence and Li [20] used a 2.5 kW high power diode laser beam (Rofin-Sinar, DL-0.25), emitting at 940 nm in order to increase wear resistance of concrete by glazing the surface using the laser interaction. Peach et al. [21,22,23] studied laser scabbling of concrete. Key factors affecting the laser scabbling of concrete were found. In addition to key factors, relationships among laser interaction time, removed volume, and surface temperatures were studied. Based on these relationships, they found the effects of the composition of concrete composites on laser scabbling behavior. However, relatively low laser power density, having a laser power of 5 kW and a spot diameter of 90 mm, was used since laser scabbling selectively removes not degraded surface volume, but degraded surface layers.

In addition to the concrete surface treatment, LAM has been used to cut concrete. Using a 3 kW Nd:YAG laser, Lenk et al. [24] cut a 70 mm concrete up to 25 mm/min. They used an airtight pressure chamber and the laser beam was applied through the chamber’s window horizontally. Layer-by-layer laser machining with mechanical removal of dross was applied to cut the concrete by Crouse et al. [1]. They presented a cutting depth of 100 mm thick concrete specimen using high-power CO_2_ and diode lasers with multiple scans. Tei et al. [25] used a multi-mode high-power laser to cut concrete with a beam diameter of 10 mm and a wavelength of 1070 nm. They cut 10 mm thick concrete with a laser power of 4 kW and a scan speed of 5 mm/s. In addition, a 100 mm thick piece of concrete was cut with a laser power of 4.5 kW, a scan speed of 5 mm/s, and 10 multi-scans. Although, this study achieved 100 mm thick concrete cutting with a multi-scan technique, the dross was removed after each laser scan. Muto et al. [26] applied a laser-based hybrid technique to cut concrete and drill rock. They used a 7 kW fiber laser with a wavelength of 1070 nm. A scanning speed of 2.5 mm/s with 14 scan passes was applied to cut the 100 mm thick plain concrete. A scanning speed of 10 mm/s with 38 passes was applied to cut the 100 mm thick heavy concrete. Nguyen Phi Long at al. [27,28] studied the removal of concrete using a pulsed laser. They tested the removal performance by irradiating the laser beam downward and upward. When the pulsed laser was irradiated in the upward direction, it was confirmed that the concrete removal performance improved with the help of gravity. A quasi-continuous (QCW) pulsed laser can be used to cut and drill without auxiliary gas. They presented that a 1.6 kW laser can drill to 20 mm thickness in 10 s. Furthermore, they demonstrated two scans with 6 kW laser power and 3 mm/s cutting speed cut, and confirmed drilling of 50 mm thick specimens. Most of the previous studies applied the multi-scan technique to cut concrete, and additional tools were required to remove dross. Furthermore, the effects of the composition variation were not studied. Therefore, the systematic study of laser parameters and composition variations on concrete cutting has not been done in detail.

This research focuses on the laser cutting of cement mortar in order to find the effect of laser parameters and compositional variations of cement-based materials on cutting characteristics. First, the material compositions and the experimental setup used in this research are described. Then, the effects of compositional variations of cement-based materials, from cement paste to ultra high-performance concrete (UHPC), on laser cutting are discussed in terms of geometrical parameters such as the kerf width and penetration depth of the workpiece. In addition to the geometrical parameters, composition changes before and after laser cutting on the surface are characterized using Energy Dispersive X-ray spectroscopy (EDX) analysis. Furthermore, cutting results are characterized depending on the laser cutting speed.

## 2. Materials and Mix Design

A series of experiments was designed to investigate the effect of laser speed on the chemical and mechanical reactions of cement-based materials with different material compositions. The materials used in this study were ordinary Portland cement, undensified silica fume (Elkem 940U, Elkem, Oslo, Norway) containing about 95 wt % of SiO_2_, silica powder with a median diameter of 3.15 μm, silica sand with two different aggregate sizes, polycarboxylate-based superplasticizer with 25 wt % solid content by weight, and water. Table 1 shows the major oxide compositions of cement, silica fume, silica powder, and silica sand. It should be noted that the sum of the oxide compositions listed in Table 1 could be less than 100% since only major compositions are listed in the table for the purpose of conciseness. Furthermore, the detailed information on the materials including the particle size distribution of the sands used for UHPC can be found in the previous study done by one of the authors [29].

Three types of cement-based materials with a specimen thickness of about 4 mm were prepared. The mix proportions of the tested series used in this study are shown in Table 2. The material series name for each is designated as follows: LP, LM, and LU series stand for cement paste, cement mortar, and UHPC, respectively. The water–cement ratio is set as a key variable for material composition for the LP and LM series. For the LM series, two distinctive series were prepared based on the amount of silica sand. For example, LM0.35 and LM1-0.35 series represent cement mortar with the same water–cement ratio set at 0.35, but the cement-silica sand ratios were set differently at 1.5 and 1.0, respectively. It should be noted that minimal amounts of superplasticizer were used for the LM series with a lower water–cement ratio in order for proper mixing. On the other hand, the amount of silica fume and silica powder were set as the material variables of the LU series. 

## 3. Experiment

The cement-based materials described in Table 2 were mixed using a laboratory planetary mixer in the following manner. For the LU series, silica fume and silica sands were first dry-mixed together for about five minutes, and then cement and silica powder were added and dry-mixing was continued for an additional five minutes in order to minimize the agglomeration of particles. Water and superplasticizer were gradually added to the dry mixture while the mixer was spinning. After the mixture started to show adequate consistency, another five minutes of mixing was performed for a uniform distribution. In the cases of the LP and LM series, all solid and liquid materials were mixed together until the mixture showed adequate consistency. The mixtures were then poured into 50 mm cubic molds for compressive tests. In order to prevent moisture loss before demolding, the casted specimens were covered with plastic sheets and stored at room temperature for 24 h. After 24 h, the specimens were demolded and cured in a 23 °C water tank. The compressive strength was evaluated by following the procedure recommended in ASTM C109, at the age of 28 days after 24 h of drying in a laboratory environment, and the results listed in Table 2 were averaged using at least three specimens for each series.

**Figure 1:**
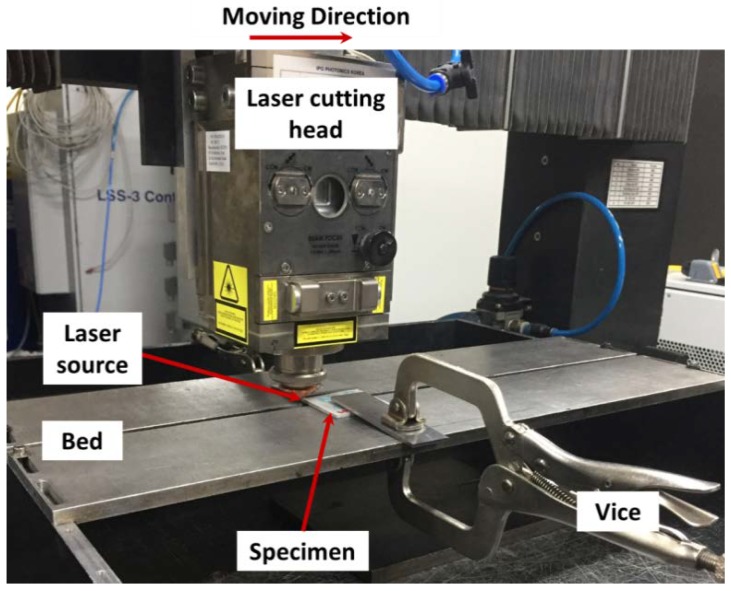
Experiment set-up.

The experimental setup is shown in Figure 1. A multi-mode continuous fiber laser (IPG YLS-10000, IPG photonics, Oxford, MA, USA) was used with a laser beam diameter of 150 μm at the focus and a wavelength of 1070 nm. In addition, the maximum available laser power of the source was 10 kW. A workpiece was placed on the bed, which consisted of two pieces with a 65 mm gap between the bed in order to provide enough space where spatter and removed materials could be ejected. The laser beam was vertically installed and irradiated on workpieces; meanwhile, the workpiece was fixed on the bed with the vice. A laser head moved in one direction while cutting. A ventilation duct to collect the dust was placed in the back of the laser cutting direction, since small size particles in the form of dust were dispersed during laser cutting. Laser power was set to 1 kW in this research, and the laser cutting speed was set to an only control parameter. After the experiments, kerf width was measured by an optical microscope. The specimens were dry-cut with a diamond saw blade to evaluate the penetration depth by optical microscope. The kerf width and penetration depth were measured from nine spots, as well as evaluated for its maximum, minimum, and average values.

The laser cutting speed was set from 4 to 14 m/min with an increment of 2 m/min. N_2_ assistant gas was applied with a pressure of 7 bar. Since line energy is an important parameter in the area of laser cutting to understand material removal mechanisms and evaluate the laser cutting efficiency, the line energy used for the experiments are tabulated in Table 3 along with the laser cutting speed. The line energy is also known as the volume energy [11].

## 4. Results and Discussion

### 4.1. Analysis of Kerf Width and Penetration Depth

#### 4.1.1. Characteristics of LP Series

**Figure 2:**
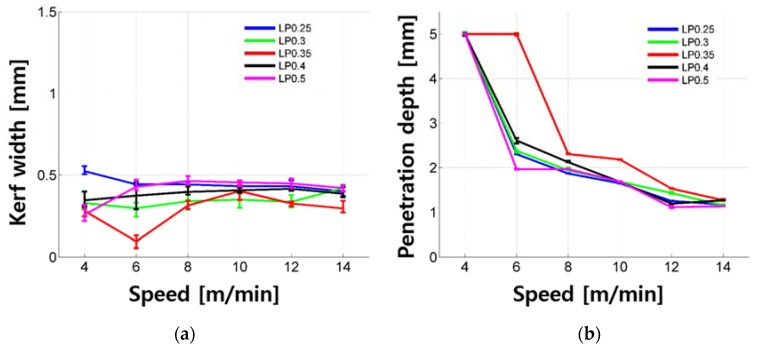
Kerf width (**a**) and penetration depth (**b**) vs. laser moving speed for the LP series.

Penetration depth and kerf width of the LP samples according to cutting speed are shown in Figure 2. When the laser cutting speed was 8 to 14 m/min, the kerf width was 0.394 ± 0.03 mm. When the cutting speed was 4 to 6 m/min, the kerf width was 0.338 ± 0.2 mm. The widths observed at high cutting speed showed a large variation. In the case of 4 m/min for each specimen, full penetration occurred. This was especially for LP 0.35, where full penetration, or complete cutting, was obtained at 6 m/min. It can be observed that the penetration depth gradually increased overall, before it was fully cut. When complete cutting occurred, the kerf width was reduced slightly. No significant trend was observed in terms of the cement to water ratio.

**Figure 3:**
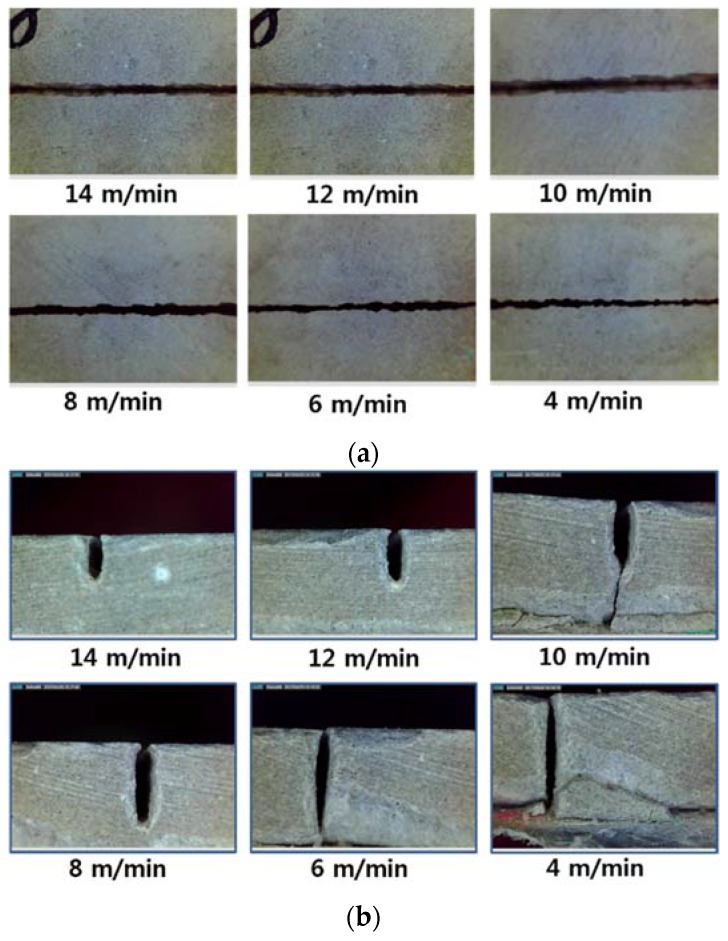
Kerf width (**a**) and penetration depth (**b**) vs. laser moving speed for LP0.35.

**Figure 4:**
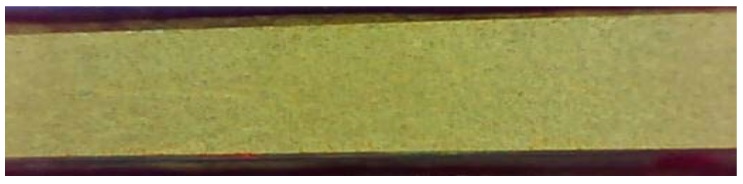
Cut surface of LP0.35 at the cutting speed of 4 m/min.

Top and cross-sectional views of the LP are shown in Figure 3. The kerf width shows an almost constant and clean cut kerf width. In addition to kerf width, the penetration depth also shows a clean material removal region whether complete cutting was achieved or not. A heat affected zone (HAZ) is seen near the inner surface of penetration hole (see Figure 4). Its length was ~0.2 mm and all LP show similar HAZs. According to the observation, overall cut quality was good since no dross, striation marks, burning, or porosity were observed, and a smooth cut edge was observed.

#### 4.1.2. Characteristics of LM Series

**Figure 5:**
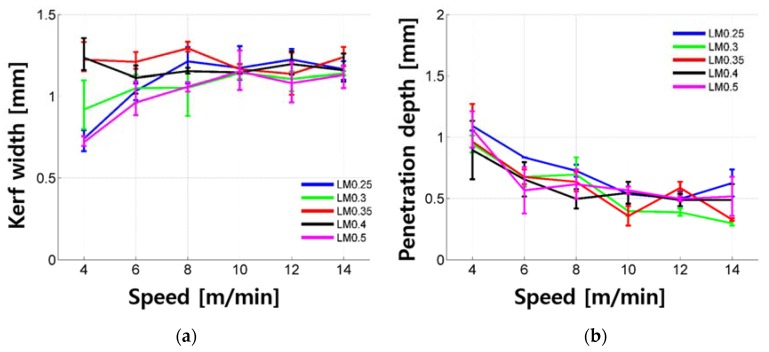
Kerf width (**a**) and penetration depth (**b**) vs. laser moving speed for the LM series.

Penetration depth and kerf width of the LM samples according to cutting speed are shown in Figure 5. The penetration depth increased exponentially as the cutting speed decreased. Under the given laser parameters, the material was removed and penetration depth can be observed for all LM samples so that the penetration depth was always greater than 0. The maximum penetration depth was 1.271 mm and the penetration depth was mostly less than 1.0 mm. As the cutting speed decreased, the kerf width decreased. Especially, the kerf width decreased significantly and its value varied between 0.682 and 1.347 mm when the cutting speed was 4 to 6 m/min. On the other hand, the kerf width varied between 0.955 and 1.347 mm when the cutting speed was 8 to 14 m/min. Under the given experimental parameters, it is assumed that the variation of kerf width increases if the penetration depth becomes deeper. For LM samples, no significant trend was observed in regards to the cement to water ratio.

**Figure 6:**
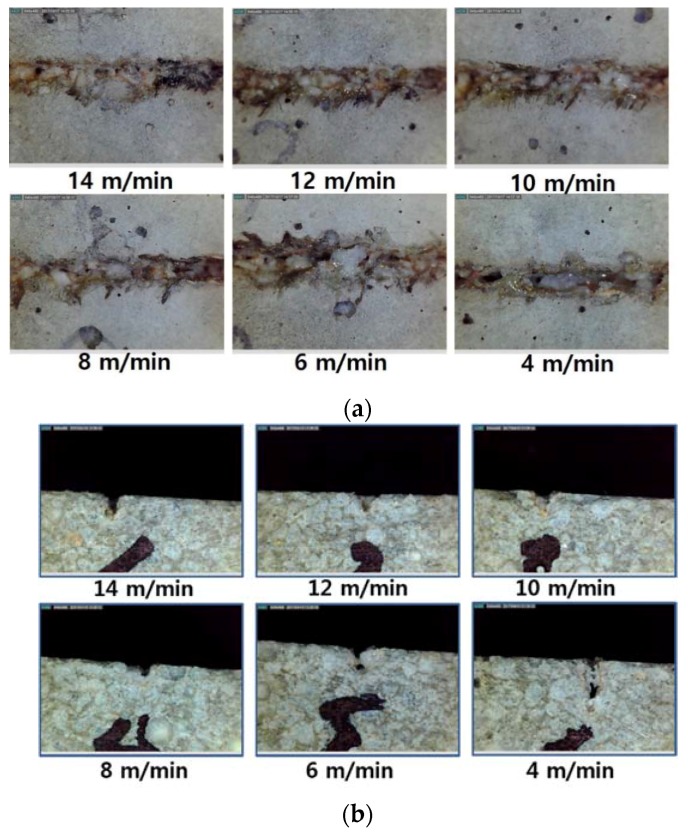
Kerf width (**a**) and penetration depth (**b**) vs. laser moving speed for LM0.4.

**Figure 7:**
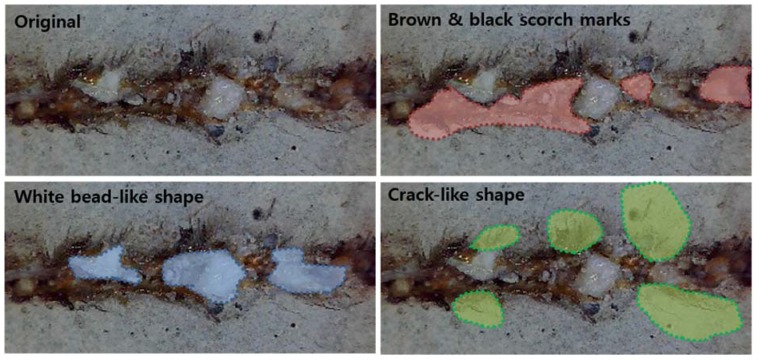
Three characteristics on the surface of the processed LM series: (1) white bead-like shape (Blue); (2) brown and black scorch mark (Red); (3) crack-like shape (Green).

Top and cross-sectional views of the LM are shown in Figure 6. For the surface of the LM after laser cutting, three characteristics can be observed: (1) a white bead-like shape; (2) a brown and black scorch mark; (3) a crack-like shape. These characteristics are shown in detail in Figure 7. The white bead-like shape seems to be formed by re-melting silica sand and cement, while water was not contained. Water may be evaporated due to the low boiling temperature of water compared to the cement or silica sand. It is assumed that the crack-like shape was formed by high pressure. The high pressure was generated from two sources: the N_2_ assistant gas and evaporation during laser cutting [30,31,32,33,34,35]. 

#### 4.1.3. Characteristics of LM1 Series

**Figure 8:**
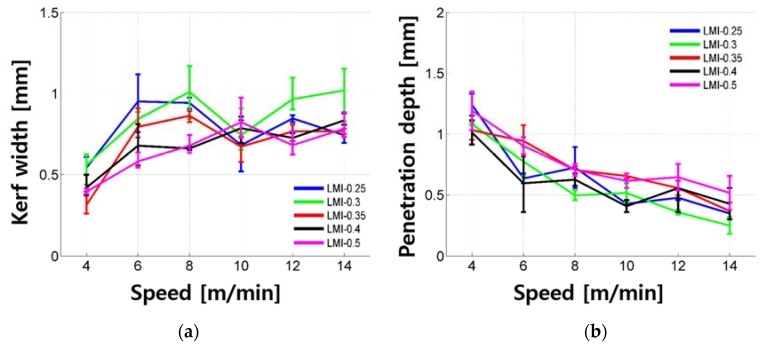
Kerf width (**a**) and penetration depth (**b**) vs. laser moving speed for LM1 series.

Penetration depth and kerf width of the LM1 samples according to cutting speed are shown in Figure 8. The penetration depth increased gradually as the cutting speed decreased. Given the fact that all LM1 samples showed penetration depth, it can be pointed out that the used laser parameters certainly provided material removal. The maximum penetration depth was 1.351 mm and the penetration depth was mostly less than 1.242 mm. As the cutting speed decreased, the kerf width decreased. Especially, the kerf width decreased significantly and most of the kerf widths were less than 0.500 mm at a cutting speed of 4 m/min. The kerf width also decreased significantly at a cutting speed of 10 m/min. For LM1 samples, it seems that LM1-0.4 and LM1-0.5 showed relatively narrower kerf widths compared to LM1-0.35 and LM1-0.25. Therefore, it may be possible that when the weight percent of silica sand is the same as that of cement, the weight percent of water may influence the laser cutting characteristics. However, further investigation is required to see this more clearly.

**Figure 9:**
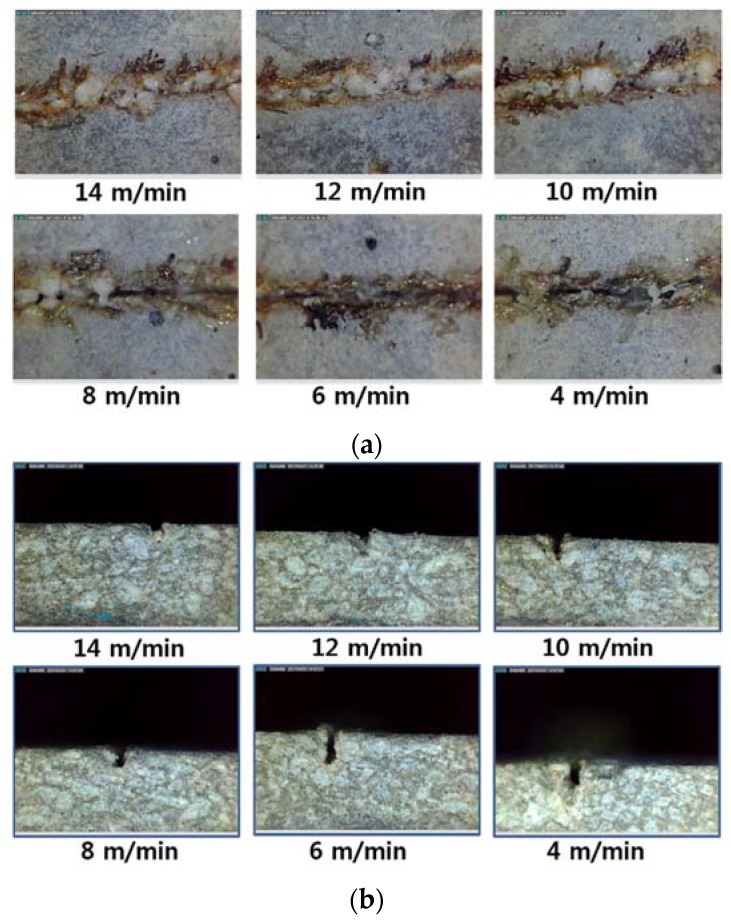
Kerf width (**a**) and penetration depth (**b**) vs. laser moving speed for LM1-0.3.

Top and cross-sectional views of the LM1-0.3 are shown in Figure 9. Uneven kerf width can be seen and material removal is inconsistent. The white bead-like shape is observed inside the kerf width similar to the LM series. It may be formed by the re-deposition of processed LM1. This white bead-like shape is observed mostly when the laser cutting speed was equal to or higher than 8 m/min. A smaller white bead-like shape and crack-like shape are observed at a laser speed of 4 to 6 m/min, while black scorch marks are clearly seen. In this speed range, deeper penetration depth was obtained.

#### 4.1.4. Characteristics of LU Series

**Figure 10:**
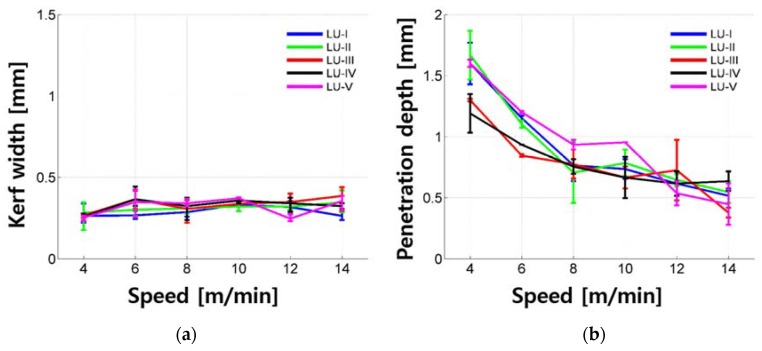
Kerf width (**a**) and penetration depth (**b**) vs. laser moving speed for LU series.

Penetration depth and kerf width of the LU series according to cutting speed are shown in Figure 10. The penetration depth increased exponentially as the cutting speed decreased. Since all LU samples tested in this study show penetration depth, it is possible that the LU was removed under the given laser parameters. The maximum penetration depth was 1.868 mm and the penetration depth was mostly less than 1.669 mm. Interestingly, the kerf width was almost constant and its value was around 0.317 mm regardless of the cutting speed. Moreover, the kerf width shows small variation and its variation is almost less than 0.100 mm, which is about one-third of that of the LM and LM1 series. No significant trend was observed regarding the amount of silica fume and silica powder. 

**Figure 11:**
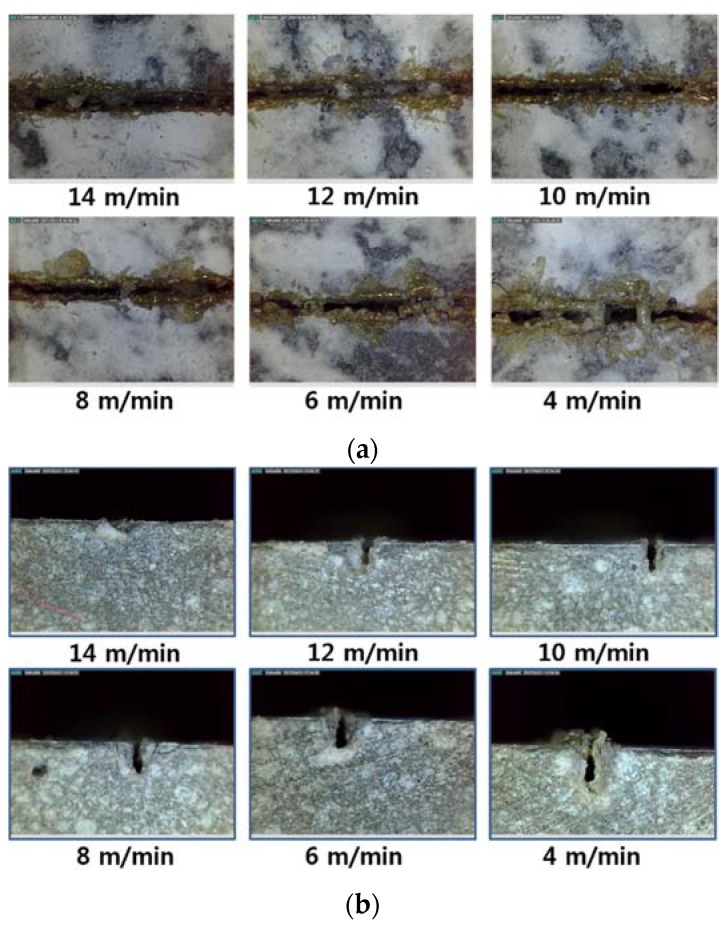
Kerf width (**a**) and penetration depth (**b**) vs. laser moving speed for LU-III.

Top and cross-sectional views of the LU series are shown in Figure 11. Different to the LM and LM1 series, almost even kerf width can be seen for LU samples. While a white bead-like shape and brown scorch marks can be observed, no noticeable crack can be detected by bare eyes. This phenomenon might be explained by the high strength of UHPC, which can resist crack opening even under high-thermal expanding pressure.

### 4.2. EDX Analysis

Through EDX analysis, the weights of chemical components constituting LM and LU-1 were observed. For the EDX analysis, LM0.4 and LU-1 were observed after characterizing the surface and setting observation points using a portable microscope (Dino-Lite, AnMo, New Taipei City, Taiwan). The concrete specimens where the surface characteristics can be observed clearly were selected. The selected specimens were cut into a proper size for the EDX analysis using a high-speed 3-mm-thick mechanical cutter (Dewalt, D28720, Dewalt, Baltimore, MD, USA.). The specimen size of LM0.4 and LU-I was 17.79 mm (width) × 5.88 mm (height) × 4.00 mm (thickness) and 16.36 mm (width) × 6.48 mm (height) × 4.00 mm (thickness), respectively. Then, the specimen surface was coated with mostly platinum (Pt) and a very small amount of zirconium (Zr). The EDX analysis was carried out with a high-resolution scanning electron microscope (Mira LMH, TESCAN, Brno, Czech Republic). The beam intensity was 20.0 kW and the working distance (WD) was 14.5 mm. At least three analysis points were repeatedly measured at each characterized surface due to the mineralogical inhomogeneities. After the EDX analysis, Pt and Zr were excluded from the results of the composition of the specimens for the composition analysis. 

**Figure 12:**
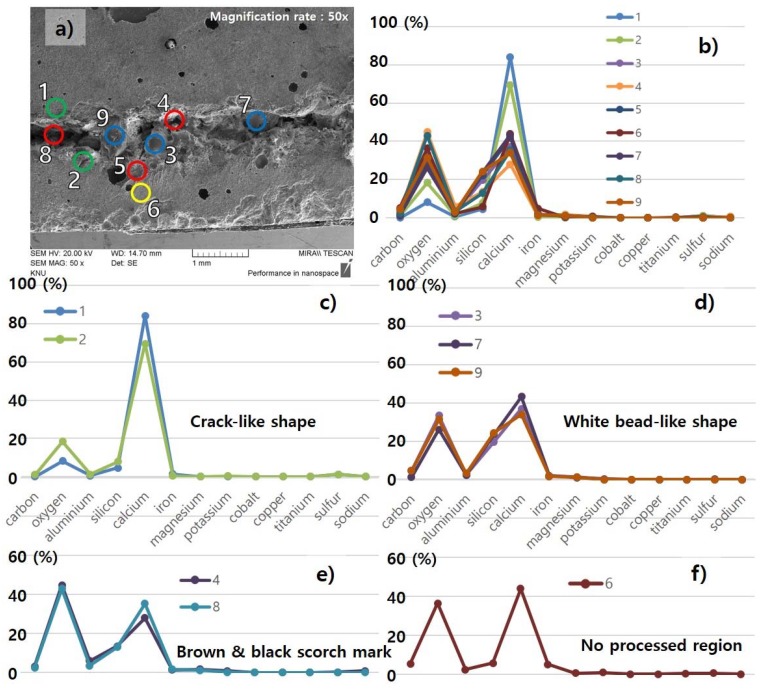
EDX analysis of LM0.4 with a cutting speed of 12 m/min, (**a**) measured points of white bead-like shape (Blue), brown & black scorch marks (Red), and crack-like shape (Green); (**b**) comparison of all measured points; (**c**) component distribution of the crack-like shape; (**d**) component distribution of the white bead-like shape; (**e**) component distribution of the brown and black scorch mark; (**f**) component distribution of non-processed region.

Three characteristics observed on the surface of the processed LM0.4 with the cutting speed of 12 m/min were investigated in detail with EDX analysis, as shown in Figure 7 and Figure 12. From the three regions from Figure 7, several points were selected and compositions at each point were measured using the EDX, as shown in Figure 12. The weight percent of the selected points for EDX analysis are tabulated in Table 4. When the specimen was first prepared, the abundant components were oxygen (36.32%) and calcium (43.74%). In addition, the LM0.4 consisted of about 5% carbon, silicon, and iron. After the laser-material interaction, the weight percent of components in the LM0.4 changed. The most significant change of the components is observed in the crack-like shape, as shown in Figure 12c. The weight percent of calcium increased to 84.13% and 69.5% at points 1 and 2, respectively. However, the weight percent of oxygen decreased to 8.1% and 18.35% at points 1 and 2, respectively. This phenomenon might be attributed to the release of gases (CO_2_, H_2_O) from dehydration of calcium hydroxide (Ca(OH)_2_) and thermal decomposition of calcium carbonate (CaCO_3_) under high temperature interaction [28]. Therefore, it should be noted that the released gases caused not only the crack opening, but chemical compositional change. At the white bead-like shape, it is clearly observable that the weight percent of silicon increased. From this observation, we expect that the weight percent of silicon increased in the form of liquid SiO_2_ from the cavity hole to the surface through the flow of thick molten pool formed by high surface temperature [27,36]. The distribution of the components’ weight percent at the brown and black scorch mark is shown in Figure 12e. The weight percent of oxygen increased and calcium decreased slightly at points 4 and 8. This is contrary to the above mentioned phenomena observed at points 1 and 2. It is possible that the released gases may not be completely removed so that the gases are trapped in this region.

**Figure 13:**
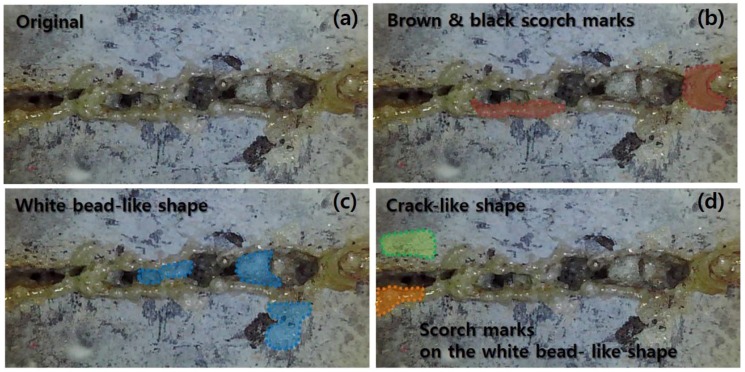
Three characteristics on the surface of the processed LU series: (**a**) no processed region; (**b**) brown and black scorch mark (Red); (**c**) white bead-like shape (Blue); (**d**) crack-like shape (Green), and scorch marks on the white bead-like shape (Orange).

**Figure 14:**
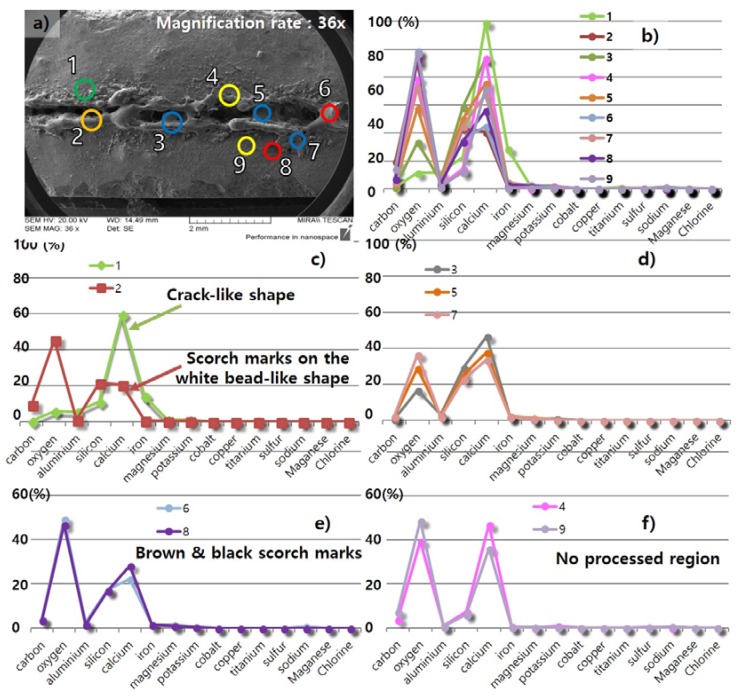
EDX analysis of LU-I with a cutting speed of 4 m/min, (**a**) measured points of white bead-like shape (Blue), brown and black scorch marks (Red), crack-like shape (Green), and scorch marks on the white bead-like shape (Orange); (**b**) comparison of all measured points; (**c**) component distribution of the crack-like shape and scorch marks on the white bead-like shape; (**d**) component distribution of the white bead-like shape; (**e**) component distribution of the brown and black scorch mark; (**f**) component distribution of non-processed region.

On the surface of LU-I, an additional characteristic was observed besides the three characteristics observed on the surface of LM0.4. Thus, four characteristics observed on the surface of the processed LU-I with a cutting speed of 4 m/min were investigated in detail with EDX analysis, as shown in Figure 13 and Figure 14. The weight percent of the selected points for EDX analysis are tabulated in Table 5. The basic constituents of LU-I was mostly composed of oxygen (39.44%) and calcium (46.73%). After laser cutting, the weight percent of the components in the LU-I changed according to the different surface shapes. The most significant change in the components was observed in the crack-like shape, as shown in Figure 14, point 1. The weight percent of calcium increased from 46.7 to 59.6% at point 1. On the other hand, the oxygen weight percentage decreased from 39.44% to 5.7%. In the white bead-like shape, an increase in the proportion of silicon was observed at points 3, 5, and 7 (Figure 14d). It is expected that the ratio of silicon component increased because the heat-affected solid SiO_2_ melted and flowed on the concrete surface. Then it solidified. As a result, the crack-like shape and the white bead-like shape of the LU-1 was similar to the LM0.4. In the brown and black scorch marks, the weight percent of oxygen increased and calcium decreased. In the crack-like shape, the emitted gas generated by laser cutting may be released through the cracks. In the brown and black scorch marks, similar phenomena were observed as shown in the LM0.4 EDX analysis. Thus, we also expect that trapped gases may result in in the brown and black scorch marks. In addition, LU-I was characterized by one additional characteristic when compared to LM0.4. It was the scorch marks on the white bead-like shape. This shape features both the white bead-like shape and brown and black scorch marks. The weight percent of silicon increased, but the weight percent of oxygen was observed to be similar to the point where no laser cutting was performed. This phenomenon is possible because the surface was burned black by heat and the gas could not be removed while the liquid SiO_2_ was solidified.

### 4.3. Comparison of Samples and Effect of Composition on Laser Cutting Characteristics

**Figure 15:**
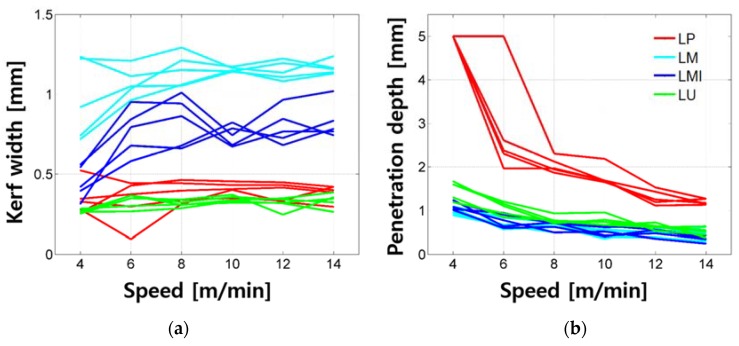
Comparison of kerf width (**a**) and penetration depth (**b**) based on the composition.

The kerf width and penetration depth of all samples tested in this research are compared and shown in Figure 15. The differences depending on the material composition mixture are clear. First, the evenness of kerf width decreases as the total quantity of silica sand increases. When comparing the quantity of the silica sand out of total weight with the same cement–water ratio, LM0.25, LM1-0.25, LU-I, and LP0.25 contain 48.7%, 38.9%, 32.0%, and 0% of the silica sand, respectively. This can be explained by understanding the laser-cement interaction proposed by Lee and Pyo [37]. Since the reflectivity of the silica sand may be higher compared to the cement particles or cement hydration products, the reflection of the laser beam by the silica sand may affect the unevenness of the kerf width. Furthermore, the larger-sized and stronger crystalline structures of silica sand compared to cement hydration products would also lead to the unevenness of kerf width. Therefore, the evenness of the kerf width decreases as the silica sand increases. Moreover, since the LU-I has smaller sized silica sand, in addition to the same-sized silica sand as LM and LM1 series, the smaller-sized silica sand may affect the less unevenness compared to the LM and LM1 series, but further investigation is required to fully understand this issue. 

Second, differences in the kerf width among the LP, LM, LM1, and LU series can be clearly observed. The LM series shows the widest kerf width and the kerf width decreases in the order of the LM1, LP, and LU series. However, there is a slight overlap between the kerf widths of the LP and LU series. The penetration depth also shows differences among the LP, LM, LM1, and LU series. The penetration depth of the LP series shows the deepest penetration depth and complete cutting achieved, and the penetration depth decreases in the order of the LU, LM1, and LM series. There is a slight overlap between the penetration depths of the LM and LM1 series. 

The LP series can be regarded as a basic cement-based material with its simple mixture. Comparing LP and LM1, when the same weight percent of silica sand and cement is added to the sample, the penetration depth decreased almost in half. However, the kerf width increased almost twice. When the weight percent of silica sand increases from 1 to 1.5%, or LM1 to LM, the kerf width increases almost twice, while the penetration depth remains the same. If silica fume and silica power are added to the LM1 or LU, the kerf width decreases almost in half and the penetration depth slightly increases.

Based on these analyses, adding silica sand into basic cement-based material results in decreasing the penetration depth in half and increasing the kerf width double. The kerf width increases due to the formation of a white bead-like shape, brown scorch marks, and crack-like shape. Furthermore, adding more silica sand, or 1.5 times more, into the basic cement-based material results in decreasing the penetration depth in half and increasing the kerf width three times. Therefore, it is confirmed that increasing the amount of the silica sand on the basic cement-based material leads to increasing the kerf width linearly and degrades the cut quality during laser cutting. In addition, if silica fume and powder were added in addition to the silica sand, it decreases the penetration depth while maintaining the kerf width with the formation of brown scorch marks.

## 5. Conclusions

To overcome the current limitations caused by the conventional cutting method, laser cutting can be a good option. The understanding of laser cutting characteristics on cement-based materials is so important to fully utilize the advantages of laser cutting on this application. Hence, the multi-mode fiber laser was applied to cut the cement-based materials and its mechanical and chemical characteristics were investigated depending on the composition variation.

1. Under the tested laser parameters, only the cement paste provides both complete cutting and good cut quality.
2. The surface of the cement mortar after the laser cutting was characterized by a bead-like shape, scorch marks, and a crack-like shape, and those characteristics were clearly observed. For the UHPC, no crack-like shape was observed by the naked eye due to the high strength of the material to resist crack opening, even under high-thermal expanding pressure.
3. EDX analysis of the cement mortar and UHPC under laser interaction revealed that chemical composition changes were caused by various mechanisms including dehydration of calcium hydroxide and thermal decomposition of calcium carbonate.
4. Adding silica sand into the basic cement-based material resulted in decreasing the penetration depth in half and increasing the kerf width double.
5. Increasing the amount of silica sand on the basic cement-based material led to increasing the kerf width linearly and degrading the cut quality during laser cutting.
6. If silica fume and silica powder are added in addition to silica sand, it decreases the penetration depth while maintaining the kerf width with the formation of brown scorch marks and a little bead-like shape.

It should be noted that a series of characterization tests on the effect of the laser on calcium-silicate-hydrate (C-S-H) and un-hydrated cement particles need to be conducted in the future as an extension of the present study for a better understanding of microscopic changes of cement-based materials under laser interaction.

## Figures and Tables

**Table 1:** Chemical compositions of the used solid constituents determined by X-ray Fluorescence (XRF) analysis.

Chemical Composition (wt %)	Cement	Silica Fume	Silica Powder	Silica Sand
CaO	62.9	0.57	0.33	0.44
SiO_2_	17.6	95.5	97.9	93.0
Al_2_O_3_	3.25	0.0	0.89	3.61
Fe_2_O_3_	8.28	0.44	0.13	0.98
MgO	0.92	0.33	0.0	0.0
SO_3_	4.12	0.79	0.0	0.0
K_2_O	2.08	1.68	0.0	1.28
P_2_O_5_	0.31	0.73	0.68	0.67

**Table 2:** Mix design of the tested cement-based materials (proportions by weight).

Series	Cement	Water	Silica Fume	Silica Powder	Silica Sand I ^†^	Silica Sand II ^‡^	Superplasticizer ^§^	Compressive Strength (MPa)
LP0.5	1	0.5						56.1
LP0.4	1	0.4						77.7
LP0.35	1	0.35						69.6
LP0.3	1	0.3						83.6
LP0.25	1	0.25						83.0
LM0.5	1	0.5				1.5		65.7
LM0.4	1	0.4				1.5		66.5
LM0.35	1	0.35				1.5		74.8
LM0.3	1	0.3				1.5	0.005	93.1
LM0.25	1	0.25				1.5	0.009	101.7
LM1-0.5	1	0.5				1.0		66.4
LM1-0.4	1	0.4				1.0		84.7
LM1-0.35	1	0.35				1.0		87.5
LM1-0.3	1	0.3				1.0	0.005	94.2
LM1-0.25	1	0.25				1.0	0.009	116.3
LU-I	1	0.25	0.25	0.25	0.30	0.70	0.009	141.6
LU-II	1	0.25	0.15	0.15	0.30	0.70	0.009	154.9
LU-III	1	0.25	0.10	0.10	0.30	0.70	0.009	113.4
LU-IV	1	0.25	0.25	0.15	0.30	0.70	0.009	162.1
LU-V	1	0.25	0.15	0.25	0.30	0.70	0.009	150.1

^†^ Aggregate fraction = 0.01~0.65 mm; median size = 0.15 mm. ^‡^ aggregate fraction = 0.03~1.1 mm; median size = 0.53 mm. ^§^ Solid content.

**Table 3:** Laser parameters used for the experiments.

Index	Speed (m/min)	Line Energy (J/m^3^)
1	14	2.43 × 10^11^
2	12	2.83 × 10^11^
3	10	3.40 × 10^11^
4	8	4.24 × 10^11^
5	6	5.66 × 10^12^
6	4	8.49 × 10^12^

**Table 4:** Composition (wt %) by EDX analysis of LM0.4 with a cutting speed of 12 m/min. Measured points are specified in Figure 12.

Point #	Carbon	Oxygen	Aluminum	Silicon	Calcium	Iron	Magnesium	Potassium	Titanium	Sulfur	Sodium
1	0	8.1	0.58	4.56	84.13	1.39	0	0	0	1.24	0
2	1.01	18.35	1.25	7.85	69.5	0.44	0	0.42	0	1.17	0
3	4.53	33.38	2.46	19.63	37.05	1.6	1.36	0	0	0	0
4	3.11	44.9	5.94	13.5	27.95	1.23	1.65	0.85	0	0.15	0.72
5	1.8	28.46	2.26	22.27	41.91	1.73	1.11	0.27	0	0.19	0
6	5.21	36.32	2.33	5.67	43.74	4.88	0.39	0.73	0.27	0.46	0
7	1.14	26.18	2.34	23.55	43.32	2.05	0.95	0.32	0	0.17	0
8	2.45	42.98	3.42	12.97	35.28	1.58	1.13	0.19	0	0	0
9	4.51	31.44	3.11	24.33	33.92	1.57	1.12	0	0	0	0

**Table 5:** Composition (wt %) by EDX analysis of LU-I with a cutting speed of 4 m/min. Measured points are specified in Figure 14.

Point #	Carbon	Oxygen	Aluminum	Silicon	Calcium	Iron	Magnesium	Potassium	Titanium	Sulfur	Sodium	Manganese
1	0.51	5.76	5.66	11.32	59.58	14.3	1	1.03	0.54	0	0	0.3
2	9.73	45.4	1.15	21.68	20.47	0.69	0.4	0.48	0	0	0	0
3	0.73	12.71	2.97	29.31	46.56	2.27	0.58	0.87	0	0	0	0
4	3.66	39.44	0.86	7.27	46.73	0.6	0.16	0.92	0	0.36	0	0
5	1.81	28.98	2.5	25.1	37.8	2.13	1.35	0.33	0	0	0	0
6	4.14	49.35	2.6	17.5	22.36	1.47	1.47	0.4	0	0	0.71	0
7	2.2	36.45	1.85	22.48	33.78	1.91	1.02	0.31	0	0	0	0
8	3.59	46.78	1.54	17	28.11	1.52	0.92	0.54	0	0	0	0
9	7.16	48.7	0.62	5.91	36	0.4	0.39	0	0	0	0.46	0
